# Macroautophagy and Cell Responses Related to Mitochondrial Dysfunction, Lipid Metabolism and Unconventional Secretion of Proteins

**DOI:** 10.3390/cells1020168

**Published:** 2012-06-20

**Authors:** Stéphane Demine, Sébastien Michel, Kayleen Vannuvel, Anaïs Wanet, Patricia Renard, Thierry Arnould

**Affiliations:** Laboratory of Biochemistry and Cell Biology (URBC), Namur Research Institute for Life Sciences (NARILIS), University of Namur (FUNDP), 61 rue de Bruxelles, Namur 5000, Belgium; Email: stephane.demine@fundp.ac.be (S.D.); sebastien.michel@fundp.ac.be (S.M.); kayleen.vannuvel@fundp.ac.be (K.V.); anaïs.wanet@fundp.ac.be (A.W.); patsy.renard@fundp.ac.be (P.R.)

**Keywords:** mitophagy, lipophagy, specific autophagy, organelle dysfunction, triglyceride accumulation, protein secretion

## Abstract

Macroautophagy has important physiological roles and its cytoprotective or detrimental function is compromised in various diseases such as many cancers and metabolic diseases. However, the importance of autophagy for cell responses has also been demonstrated in many other physiological and pathological situations. In this review, we discuss some of the recently discovered mechanisms involved in specific and unspecific autophagy related to mitochondrial dysfunction and organelle degradation, lipid metabolism and lipophagy as well as recent findings and evidence that link autophagy to unconventional protein secretion.

## 1. Introduction

Autophagy is a complex and highly regulated cellular degradation pathway, well conserved among eukaryotes. Cellular components that are ultimately hydrolysed by lysosomes are used for energy production or recycled. Three different types of autophagy have been described so far: (1) microphagy, (2) chaperone-mediated autophagy and (3) macroautophagy. Microautophagy is mainly activated either by rapamycin or nitrogen starvation, even if these conditions are also known to stimulate macroautophagy. In the microautophagy process, proteins are directly imported into lysosomal lumen by invagination of the membrane of this organelle. This form of autophagy has recently been reviewed in detail [1]. In the second process, proteins containing a KFERQ motif are directly recognized by a set of chaperone proteins including Hsp70 (Heat-Shock Protein 70) that target the proteins to lysosomes where it is internalized upon interaction with LAMP-2 (Lysosomal Associated Membrane Protein 2) and ultimately degraded into lysosomal lumen [2].

The content of this review considers only some aspects of macroautophagy (hereafter autophagy) in which cytoplasmic components or organelles (the cargos) are engulfed into specialized vesicles called autophagosomes, which mature and ultimately fuse with lysosomes, forming autophagolysosomes in order to degrade their content.

While a continuous and dynamic process, autophagy can be mechanistically divided into five separate steps: (1) initiation of autophagosome formation, (2) nucleation (3) elongation, (4) maturation and (5) degradation of autophagosome. The first step requires the assembly of a complex composed of Ulk1 (Unc-51-like kinase), Atg13 (Autophagy-related protein 13), FIP200 (Focal adhesion kinase (FAK) family Interacting Protein of 200 kDa) and Atg101 (Autophagy-related protein 101). This process can be inhibited by mTORC1 (mammalian Target Of Rapamycin Complex 1) protein complex comprising Raptor (Regulatory Associated Protein of TOR), mLST8/GβL (mammalian Lethal with Sec13 protein 8/G protein β-subunit-like protein), PRAS40 (Proline Rich Akt Substrate 40) and Deptor (DEP-domain-containing and mammalian Target of Rapamycin-interacting protein) [3,4]. Interaction with Atg5 and Atg12 is also needed for initiation of autophagosome formation. The next steps are the nucleation and elongation of autophagosome membrane. The activation of a pro-autophagic complex composed of Beclin1/PI3K (Phosphatidyl Inositol 3-Kinase) and subsequent recruitment of Atg (autophagy-regulated proteins) (see glossary of autophagy-related molecules and processes [5]) are crucial for the formation of autophagosomes. The formation of Beclin1/PI3K complex can be inhibited by Bcl-2 and is stimulated by UV irradiation Resistance-Associated tumor suppressor Gene (UVRAG). The activation of PI3K in autophagy is mediated by Ambra1 protein, Vps34 (Vacuolar Protein Sorting 34), Atg14 and a few other less characterized proteins. Second, elongation of autophagosome membrane requires the assembly of two ubiquitin-like systems. In the first system, Atg12 is conjugated to Atg5 after interaction with both Atg7 and Atg10 (E1/E2-like enzymes). Atg16L is finally recruited to form a complex with Atg5/Atg12. The second ubiquitin-like complex is required for maturation of LC3b. This protein is synthesized as a propeptide that is cleaved by Atg4 in order to form LC3-I. The mature form of LC3, LC3-II, is generated by conjugation of a phosphatidylethanolamine, a reaction catalysed by Atg7/Atg3, and then inserted into the autophagosomal membrane. After complete engulfment of cargo and formation of autophagosome, the vesicle will maturate by fusion with lysosomes in order to degrade its content. For more details related to autophagosome formation [6], molecular mechanisms and signalling/regulatory pathways of autophagy, we refer to recent reviews [6,7,8].

Implications of autophagy have been discovered in numerous cellular processes. First, autophagy is notably involved in quality control of both proteins and organelles. Second, this pathway is also known to regulate cellular differentiation [9] and plays an important role in development and survival in the very first hours following birth that exposes the individual to a brief nutrient starvation condition. Third, both innate and adaptive immunity and inflammation are also directly regulated by autophagy [10], especially in response to bacteria invasion [11]. If autophagy is often described as a pro-survival pathway as it allows recycling of cellular elements, damaged or not, and energy production facing nutrient deprivation, it can also directly positively or negatively regulate several forms of cell death including apoptosis, necrosis, necroptosis, and pyroptosis [12]. In addition, the role of autophagy in cell death has recently been challenged as in a large chemical screen not a single compound induced cell death by autophagy [13]. However, arguments to rule out the participation of autophagy to cell death in this study can be challenged as the knockdown of Atg5/Atg7, failing to prevent and rather accelerating chemotherapy-induced cell deaths, is indirect at best and because chemotherapeutical compounds are known to induce multiple routes of programmed cell death. Therefore, it is sometimes very difficult to evaluate the role of autophagy as a cell death or a cell pro-survival process. Autophagy was also presented as a key process in cellular aging, in tumorigenesis or cancer drug resistance, process and control of autophagy being often presented as a novel cancer treatment. All these roles have been comprehensively reviewed in recent papers [14,15].

In this review, we will focus on some novel roles and mechanisms described for autophagy. First, we will investigate the involvement of autophagy in the regulation and the degradation of mitochondrial population, a process called mitophagy. We will discuss the molecular mechanisms of mitophagy but also the interplay between this process and retrograde communication with other damaged organelles such as endoplasmic reticulum or lysosomes. In the second part of this review, we will overview the most recent forms of specific-autophagy: lipophagy, an autophagic process that specifically targets lipid droplets. The first discovered elements of molecular machinery of this process will be discussed as well as the more general role of autophagy in lipid metabolism, in adipocyte biology and in some lipid-related troubles such as obesity or alcohol-induced fatty liver. Finally, we will discuss the implication of autophagy in unconventional secretion of proteins. Indeed, if autophagy is essentially involved in degradation and recycling of molecules and organelles, it also seems to play a role in non-degradative pathways such as unconventional secretion of proteins.

## 2. Autophagy and Mitochondrial Dysfunction

Autophagy was first considered as a regulated non-selective catabolic process that recycles intracellular components to compensate for nutrient deprivation and ensures, accidentally, degradation of organelles. However, more recently, accumulating data have highlighted the existence of specific forms of autophagy targeting various cargos including organelles: mitochondria (mitophagy) [16], endoplasmic reticulum (reticulophagy) and ribosomes (ribophagy) [17], peroxisomes (pexophagy) [18] and lipid droplets (lipophagy) [19,20,21] but also invading bacteria (xenophagy) [22]. Over the past few years, mitophagy mechanisms (regulators and effectors) have been extensively studied and will be first described in the next section that will also summarize emerging data on the reciprocal crosstalk between mitochondria and autophagy.

### 2.1. Molecular Effectors and Mechanisms of Regulation Involved in Mitophagy

**Figure 1:**
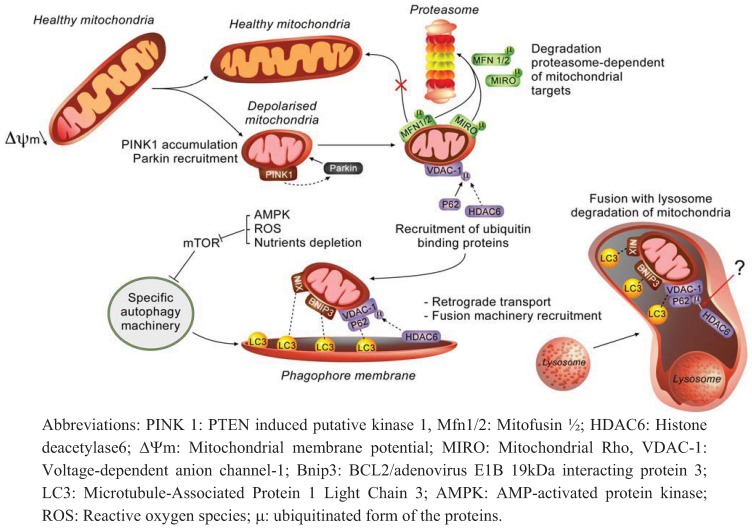
Mechanisms involved in mitophagy.

Mitophagy can be defined as the specific degradation of mitochondria by autophagy. In recent years, mitophagy has been well documented and accumulating data now demonstrate the importance of this mitochondrial quality control to preserve correct organelle function and morphology [23]. General steps of this catabolic process share similarities with non-selective autophagy as mitochondria are first engulfed by double membranes that lead to autophagosome formation before the delivery to lysosomes for hydrolytic degradation and recycling (Figure 1). However, this process requires additional molecular actors and regulation steps to selectively recognize its cargo and to recruit autophagy machinery to specifically degrade mitochondria. The PTEN-induced putative kinase 1 (PINK1) and the E3-ubiquitin ligase Parkin, both associated with familial form of Parkinson disease (PD), were the first molecules to be related to mitochondrial integrity maintenance in flies [24,25] and then to mitophagy in mammals [26,27,28]. Under basal condition, PINK1 abundance is kept very low as once imported at the inner mitochondrial membrane and processed by the mitochondrial processing peptidase (MPP) and/or presenilin-associated rhomboid-like protease (PARL), the kinase is rapidly degraded by a MG132-sensitive protease [29,30]. However, if mitochondrial membrane potential is compromised or artificially disrupted by uncoupling molecules such as carbonyl cyanide-*m*-chlorophenyl hydrazone (CCCP), PINK1 accumulates at outer mitochondrial membranes as a result of potential-dependent import default [31,32]. Even if the nature of the interaction between PINK1 and Parkin remains to be determined, it has been shown that PINK1 activity is required to trigger Parkin translocation to outer mitochondrial membrane [33]. In addition, these authors have shown that the overexpression of the kinase is sufficient for Parkin accumulation, even in the absence of mitochondrial membrane potential uncoupling [33]. Nevertheless, mitochondrial membrane potential modification seems to act as a signal to report organelle “health” status and the protein pair PINK1/Parkin is the sensor that regulates the recruitment of autophagy machinery to degrade damaged mitochondria. Indeed, «unstable» mitochondrial membrane potential (Δѱm) and redox transitions may occur as a result of diverse pathological states that impair ROS [34] and/or calcium [35] homeostasis.

Once translocated to mitochondria, the E3-ubiquitin ligase activity of Parkin plays a central role in mitophagy as revealed by the phenotypes resulting from the overexpression of different PD-related Parkin mutants, that block mitochondria degradation at distinct steps [36]. Indeed, depending on the nature of ubiquitination, different proteins can be recruited and engaged at multiple steps of the process. The identification of targets ubiquitinated by Parkin gave progressively clues about how this process is regulated (Figure 1). For example, Geisler and colleagues reported that the lysine 23 (K23) poly-ubiquitination of VDAC-1 upon a CCCP-treatment allows the binding of p62 (also known as SQSTM1) [33]. Two domains of this adaptor protein, the ubiquitin-associated domain (UBA) and the LC3-interacting region (LIR), facilitate the subsequent recruitment of autophagosome formation machinery at depolarised mitochondria [33]. However, despite being extensively illustrated in both the mitophagy model and pexophagy models, the implication of p62 in Parkin-mediated mitophagy is still controversial as some studies confirmed the implication of p62 [33,36] while other studies did not show any impact of VDAC-1 or p62 knockout on mitophagy in mouse embryonic fibroblasts (MEFs) [37].

While cycling between fusion and fission, a partial depolarised area of the mitochondria is isolated from the network by fission. Low mitochondrial membrane potential of isolated organelle leads to PINK1 accumulation, cytosolic Parkin recruitment to mitochondria and ubiquitination of various mitochondrial proteins. On the one hand, proteins such as Mfn1/2 and Miro are tagged for degradation in a proteasome-dependent manner to prevent fusion with healthy mitochondria and anterograde transport, respectively. On the other hand, ubiquitination of targets (e.g., VDAC-1) triggers ubiquitin-binding proteins recruitment such as p62 and HDAC6. Adaptor proteins such as p62 trigger mitochondrial priming by interacting with LC3. Other mitochondrial proteins such as Nix and Bnip3 also interact with LC3 to recruit phagophore membrane and promote mitochondria engulfment (the link between HDAC-6 and ubiquitinated VDAC-1 indicated by an arrow and a question mark indicates that the nature of the link is still hypothetical). HDAC6 participates at other steps of mitophagy: mitochondrial retrograde transport and fusion of autophagosome with lysosomes. Finally, mitochondria are degraded by lysosomal enzymes.

In addition to VDAC-1, the proteins Mitofusin 1 and 2 (Mfn1/2), major dynamin-related proteins involved in the fusion events of the organelle [38], are also ubiquitinated after Parkin translocation to mitochondria. However, Mfn1/2 K48-ubiquitination does not lead to the recruitment of p62 but instead enhances the proteasomal degradation of these proteins in a p97-dependent manner and thus prevents further fusion of depolarised mitochondrial segment with mitochondria showing a polarized mitochondrial membrane potential [39]. In these conditions, degradation of Mfn1/2 that impacts fusion events, highlights the importance of mitochondrial morphology and dynamic in the regulation of mitophagy. Indeed, it has been shown that mitochondrial fission generates two unequal daughter organelles regarding their membrane potential, isolating depolarised mitochondria to be degraded from the “healthy” network [40]. These findings suggest that mitochondrial morphology regulation is connected to the control of mitophagy.

In addition, several other proteins of the outer mitochondrial membrane are also K48-ubiquitinated by Parkin and degraded in a proteasome-dependent manner such as TOM20, TOM70, Fis1, Bax and Miro (Figure 1) [41,42]. In neurons, Parkin-mediated Miro ubiquitination and subsequent degradation by proteasome regulates mitochondrial trafficking and axonal transport [42]. Indeed, Wand and colleagues have shown that the rho-like GTPase Miro is phosphorylated by PINK1 and degraded by the proteasome in a Parkin-dependent fashion [43]. As Miro is known to participate in mitochondrial movement/kinesis within the cells, its degradation prevents organelle anterograde transport by releasing kinesin from its surface. On the contrary, retrograde transport of depolarised mitochondria after Parkin ubiquitination activity is mediated by HDAC6 (Histone Deacetylase 6) [36]. Indeed, it has been suggested that HDAC6, another ubiquitin-binding protein, also participates in the transport of Parkin-bound mitochondria as damaged fragments of the organelle do not accumulate at nuclear periphery in HDAC6-deficient MEFs, a transient event that is required for further fusion with lysosomes [36]. In addition, these authors have also shown that HDAC6 recruits a cortactin-dependent actin remodelling machinery to facilitate the fusion between autophagosomes and lysosomes and the subsequent degradation of their cargos [44].

In conclusion, accumulating data now evidence that Parkin, even in cell types other than neurons, is a central actor in mitophagy, regulating several aspects of the degradation process by targeting the ubiquitination of multiple mitochondrial proteins. However, as Parkin overexpression, a condition that stimulates mitophagy [33], is often used as a strategy to identify Parkin targets, the patho-physiological relevance of these substrates must be confirmed with endogenous Parkin level *in vivo*.

Beside the Parkin/PINK1 model of mitophagy, the BH3-only protein Nix (also known as Bnip3L) has been associated with the total clearance of the mitochondrial population during reticulocyte maturation [45,46]. As Atg32 (a yeast protein sharing UBA and LIR domains with the Nix protein), Nix may trigger phagophore elongation by interacting with LC3 by its LIR (LC3-interacting region) domain [47]. Nix-mediated mitophagy has been reviewed elsewhere [48] and will thus not be further developed here. Similarly, Bnip3 is also able to interact with LC3 and is required for mitophagy under hypoxia [49] and in Bax/Bak-deficient MEFs [50]. It is worth mentioning that in addition to the reticulocyte maturation model, Nix has also been reported to contribute to mitophagy in CCCP-treated HeLa cells and MEFs [51]. Indeed, these authors gathered together the two current models of mitophagy and proposed that Parkin would only trigger damaged mitochondria recognition without affecting autophagy initiation, while Nix would participate at both mitochondrial priming and autophagy initiation [51], confirming Nix requirement for membrane potential loss during mitophagy [45]. It is interesting to mention that Bnip3, sharing 56 % of homology with Nix, is also able to impair mitochondrial membrane potential that triggers mitophagy [50]. In MEFs deficient in Bax/Bak proteins, Bnip3 increases proteases-dependent degradation of both nuclear and mtDNA-encoded proteins involved in the oxidative phosphorylation, thereby reducing membrane potential that triggers mitochondrial removal by mitophagy [50].

During both selective and non-selective autophagy, mitochondrial morphology is an additional important regulator of organelle degradation fate. Indeed, while fission and mitochondrial network fragmentation is required to isolate depolarised organelle during mitophagy [40,52], Gomes and colleagues have reported that mitochondrial network elongates during starvation-induced autophagy [53]. Indeed, while cycling between fusion and fission under basal condition, reticulation of mitochondrial network results from uncompensated fusion as under starvation condition, increased cAMP level activates PKA that, in turn, phosphorylates Drp1 (dynamin-related protein 1), preventing the re-localization of the mitochondrial fission modulator to mitochondria, and thus inhibiting organelle fission. In addition, cell death is prevented because mitochondrial elongation is accompanied by increased cristae surface, ATP synthase subunit oligomerization and enhanced ATP production efficiency [53]. Thus, both selective and non-selective autophagy relies on opposite regulation of mitochondrial dynamics and morphology to preserve mitochondrial function, homeostasis and thus cell survival pathway.

The integrity of other organelles seems also to be required for proper mitophagy to occur. Indeed, lysosomal and endoplasmic stresses have been shown to impair mitochondrial removal by mitophagy.

Lysosomes are essential for autophagy, and autophagic clearance of dysfunctional mitochondria represents an important element of mitochondrial quality control [54]. It is easy to speculate that dysfunction of this organelle, such as in lysosomal storage diseases (LSD), leads to the abnormal accumulation of non-hydrolysed autophagy cargos such as mitochondria. Indeed, accumulation of abnormal and dysfunctional mitochondria has been reported in various type of LSD without real mechanistic description of mitophagy defect [55]. Mechanisms of mitochondrial dysfunction in LSD have been recently uncovered by Ballabio’s lab in a study revealing that mitochondrial accumulation is not only the result of impaired lysosomal degradation function but is also due to impaired targeting of mitochondria. Indeed, in brain of mice affected by a multiple sulfatase deficiency (MSD), reduced mitochondrial “priming” leads to the accumulation of abnormal mitochondria, leading ultimately to cytochrome c release and apoptosis [56]. Thus, impaired mitophagy may be important in the pathogenesis of LSD and it has been proposed that the chronic disruption of lysosomal function leads to the accumulation of abnormal mitochondria and promotes cell death by apoptosis [56,57]. In addition to lysosomal dysfunction in LSD, a mutation associated with an autosomal recessive form of Parkinson’s disease (PD) in the gene encoding a lysosomal P-type ATPase of unknown function (*ATP13A2*) has been also associated with autophagy defect [54]. Indeed, ATP13A2-deficient cells present increased mitochondrial fragmentation associated with enhanced ROS production and decreased autophagy flux as a result of mTOR activation [54].

ER stress can also induce mitochondrial stress, resulting in an energetic deficit, mitochondrial fragmentation and subsequent mitophagy of damaged mitochondria. In their study, Bouman and co-workers showed that Parkin expression increases in response to ER or mitochondrial stress through the specific PERK/ATF4 pathway of the unfolded protein response (UPR) [58]. Moreover, the cytoprotective function of ATF4 relies on its binding to a CREB/ATF binding site within Parkin promoter, thereby increasing its expression in response to ER stress. However, the precise role of Parkin in the communication between the ER and mitochondria is not well established and the consequences on mitophagy have not been fully addressed as yet. In addition, interconnection of mitochondria and ER at specialized domains such as mitochondria-associated ER membranes (MAMs) are well described and constriction function of ER tubules has been reported to occur during mitochondria division [59]. Interestingly, disrupting interactions between both organelles as observed in *Mfn2* knockout MEFs prevents the formation of autophagosomes during starvation, highlighting the importance of ER-mitochondria contacts during starvation-induced autophagy [60]. These authors also elegantly demonstrated that fluorescent lipid NBD-PS (NitroBenzoxaDiazol-Phosphatidyl-Serine, a molecule converted to NBD-phosphotidylethanolamine in mitochondria) transfers from mitochondria to autophagosomes and thus mitochondria could be membrane providers for autophagosome formation [60]. Further evidence of ER-mitochondria crosstalk regarding calcium storage and phosphatidylethanolamine (PE) synthesis in autophagy regulation will be also discussed later.

In summary, several studies have led over the last few years to better understanding of the mechanisms of mitochondrial removal by mitophagy (Figure 1). However, if mitochondria appear at first sight as just a substrate of autophagy, accumulating data now tend to demonstrate that the crosstalk between mitochondria and autophagy is more complex. In the next section, we will focus on this new interesting inter-relationship between mitochondria and autophagy machinery, focusing first on the importance of autophagy to preserve mitochondrial homeostasis and then, conversely, on the mitochondrial contribution to autophagy regulation.

### 2.2. How does Autophagy Preserve Mitochondria Activity?

The most obvious link between autophagy and mitochondria is thus mitophagy, a specific process of mitochondria removal that controls the turn-over of damaged organelles and preserves mitochondrial morphology and function. Mitophagy is also crucial at specific developmental steps that require total elimination of the mitochondrial pool such as during reticulocyte terminal differentiation in mammals [61]. Therefore, it is not surprising that impaired regulation of autophagy leads to accumulation of abnormal mitochondria [62,63,64,65]. Dysregulation of mitophagy has also been associated with the pathogenesis of neurodegenerative diseases such as Parkinson disease [66,67,68], Alzheimer disease [69] and several syndromes associated with mtDNA defects. Indeed, in primary fibroblast cultures of patients harbouring the A8344G MERRF (Myoclonic Epilepsy with Ragged-Red Fibres) mutation, mitochondrial dysfunction and oxidative stress were associated with increased mitophagy of impaired organelles [70]. The same laboratory also reported similar results for another mitochondrial myopathy, MELAS (Mitochondrial Encephalomyopathy, Lactic Acidosis, and Stroke-like episodes), associated with the A3243G mutation within mtDNA [71]. However, in these conditions, mitochondrial degradation was associated with an accumulation of autophagosomes, which suggests incomplete mitophagy [71]. However, in cybrid cells harboring mtDNA mutations/deletions, membrane-potential dependent Parkin recruitment at outer mitochondrial membrane is not sufficient to trigger mitophagy of mitochondria that display a dysfunction, as this process also requires the general induction of autophagy triggered by mTORC1 inhibition [72]. These findings confirmed previous results obtained by Ding and collaborators who showed that, in HeLa cells, Nix promoted CCCP-induced mitochondrial depolarization and reactive oxygen species generation, which inhibited mTOR signalling and activated autophagy [51]. Thus, even if it is well accepted that PINK1 and Parkin do play a role in different steps of mitophagy, their recruitment to depolarized organelles is required but is not sufficient for mitophagy completion. Besides, the importance and the necessary role of Parkin has been emphasized as, in cells with down-regulated Parkin, depolarized mitochondria are not degraded anymore when autophagy is initiated by mTOR inhibition by rapamycin [72].

Finally, two other surprising impacts of autophagy on mitochondria have been described in different cancer cells [73] and innate immune response [74]. In agreement with the reverse Warburg model, in cancer-associated fibroblasts, autophagy is increased to fuel epithelial cancer cells with recycled nutriments, thereby preserving mitochondrial activity and promoting tumour growth and metastasis through a vicious cycle of catabolism in the tumor stroma and anabolic tumour cell expansion [73,75]. This biological process has been associated with caveolin-1 loss in fibroblasts and increased expression in plasminogen activator inhibitor type 1 and type 2 (PAI-1 and PAI-2) that promote increased mitochondrial abundance and activity in cancer cells as well as reduced apoptosis [76]. A better understanding of the relationship between mitochondrial activity and autophagy between stromal and cancer cells would thus represent an interesting opportunity for therapy.

Autophagy integrity is also important for mitochondria during innate immune response as, in LPS and ATP-stimulated macrophages, depletion of LC3 and Beclin1 promotes mitochondrial dysfunction and cytosolic translocation of mtDNA in a NALP3 inflammasome in a ROS-dependent manner. These results suggest that autophagic proteins regulate NALP3-dependent inflammation by preserving mitochondrial integrity [74].

### 2.3. How do Mitochondria Contribute to Autophagy Regulation?

Even if elongation membranes mechanisms are well described, the origin of autophagosome membranes are only partly understood in terms of higher eukaryotes. Different studies have shown that plasma membrane, Golgi apparatus, endosome compartment and endoplasmic reticulum and even mitochondria might be potential sources for autophagosome formation (for interested readers on mechanisms of autophagosome biogenesis and cargo selectivity see [77]). Hailey and colleagues used live cell imaging to follow compartments-specific markers of potent sources of autophagosome membranes. Among endoplasmic reticulum, Golgi apparatus, endosomes and plasma membrane markers, only YFP-tagged cytochrome b5 (specific to mitochondria) abundantly co-localizes with LC3 when NRK58B cells were starved for 2 h [60]. Enhanced mitophagy has been excluded in this condition as only outer mitochondrial membrane proteins co-localize with LC3 whereas neither matrix nor inter-membrane space proteins did. Fluorescence signal extinction of newly formed autophagosomes after YFP-cytochrome b5 photobleaching gave further evidence of potent exchanges between outer mitochondria membrane and newly formed phagophores, at least in the starved NRK58B cell model [60], but mitochondria as providers for the formation of autophagosomes still need to be demonstrated in other models to determine whether or not it is a specific or generic process. However, regarding the crucial role of phosphatidylethanolamine (PE) in autophagy and considering both endoplasmic reticulum and mitochondria as sources of this phospholipid, both compartments might participate in the biogenesis of autophagosomal membrane and further investigations are needed to decipher events that determine the origin of membranes. In addition to a potent mitochondrial membrane supply for the biogenesis of autophagosomes, mitochondrial Bcl-2 has been proposed to regulate autophagy initiation by partially controlling Ambra1 localization, a protein that interacts with Beclin-1 and acts as a positive regulator of autophagy [78]. Even if Ambra1 is mainly cytosolic, a substantial proportion of the protein pool also localizes at mitochondria where it interacts with Bcl2 to inhibit Beclin1-dependent autophagy initiation. These authors proposed that upon autophagy induction, this interaction is disrupted and allows Ambra1 to compete with mito- and ER-Bcl2 for Beclin1 binding and thus to initiate autophagy [78]. Interestingly, it has also been shown that Ambra1 is recruited to perinuclear depolarized mitochondria, interacts with endogenous Parkin and is critical for clearance of damaged organelles after prolonged mitochondrial depolarization [79].

Both endoplasmic reticulum and mitochondria are also important intracellular calcium stores. Indeed, while the role of ER in calcium storage is widely accepted, the physiological contribution of mitochondria was debated because of its low-affinity calcium uptake [80]. The rate-limiting step of mitochondria calcium uptake is crossing the inner mitochondrial membrane by the mitochondrial calcium uniporter (MCU) subunits of the voltage-dependent MiCa calcium channel which has been recently identified [81]. In addition, it has been shown that ER to mitochondria calcium transfer is achieved at specific microdomains where both organelles are in close contact and rate limiting MCU uptake is overstepped by important IP3R-mediated calcium release [82,83]. Such interactions are crucial for mitochondrial metabolic function and cellular energetics as activity of key enzymes such as pyruvate-, α-ketoglutarate- and isocitrate- deshydrogenase are regulated by calcium [84]. Therefore, decreased calcium concentration within the mitochondrial matrix impaired ATP production and triggered activation of the cell energy sensor AMPK that has been described to promote autophagy by mTOR inhibition [85].

Redox signalling has been extensively linked to autophagy (reviewed in [86]) but the specific contribution of mitochondria (a major site of reactive oxygen species (ROS) production) to the redox control of autophagy is less clear. What we know is that starvation-induced ROS accumulation is necessary for autophagosome formation as H_2_O_2_ has been shown to regulate Atg4 protease activity through a conserved cysteine residue and thus controls LC3 lipidation and recycling [87]. However, even if these authors showed that DCFH-DA (2',7'-dichlorofluorescein-diacetate, a fluorescent probe to quantify intracellular H_2_O_2_) and mitochondrial staining co-localized in this condition [87], the direct contribution of mitochondrial ROS was not fully addressed. In HeLa cells and MEFs, it has been shown that, very rapidly after a CCCP-treatment, Nix is required for autophagy initiation by mitochondrial membrane depolarization and ROS production that inhibits mTOR [51]. In addition, in starved HeLa cells, Chen and colleagues have also reported that the starvation-induced ROS generation (and especially mitochondrial O_2_^.−^) was required for autophagy induction as overexpression of mitochondrial matrix superoxide dismutase 2 (MnSOD/SOD2) inhibits autophagy [88]. Further evidence of mitochondrial ROS contribution to autophagy regulation has also been shown by the same group as the increase in ROS levels by mitochondrial complexes I and II inhibitors is sufficient to trigger autophagy [89]. Furthermore, mitophagy associated with reduced mitochondrial respiration and increased oxidative stress is abolished by antioxidant treatment [71]. Finally, Prohibitin 1, a protein known to preserve mitochondrial respiration, has been shown to modulate TNFα-induced autophagy, a condition previously associated with a strong increase in mitochondrial ROS production [90]. Conversely, Jiang et al. (2011) used cytochrome c-deficient HeLa cells (obtained by shRNA) to question mitochondrial ROS requirement for autophagy [91]. In their model neither O_2_^− ^nor H_2_O_2_ seems to be required for staurosporine-induced autophagy as LC3 lipidation and mitochondria degradation by mitophagy were observed in cytochrome c-depleted HeLa cells without any ROS detection [91]. Although these authors described similar observation for rho^0^ HeLa cells, it is difficult to conclude regarding these results as other studies show either an increased production of ROS in rho^0^ HeLa cells compared with parental cell line [92] or a decreased production of ROS [93,94]. Thus, in conclusion, mitochondrial ROS contribution to autophagy regulation is still a debated question and result discrepancies might depend on the nature of the autophagy inducers.

Conversely, autophagy is also known to regulate oxidative stress as mitochondrial mass and ROS production is increased in FIP200 (200-kDa FAK-family interacting protein that plays important roles in mammalian autophagy) deficient hematopoietic stem cells (HSC) [95]. In addition, the inhibition of mitophagy in PINK1 knockout dopaminergic neurons increases mitochondrial O_2_^−^ production [96]. Thus, even if direct implication of mitochondrial ROS on autophagy is not clear, the later process needs to be tightly regulated as defective autophagy will lead to excessive accumulation of defective mitochondria and enhanced ROS production, further increasing mitochondrial and cell dysfunction.

As we have seen, it is well accepted that mitochondria with reduced membrane potential are targeted by mitophagy. However, the consequence of mitochondrial DNA (mtDNA) alterations (mutations, deletions, *etc.*) on autophagy is still poorly described. Gilkerson and colleagues have shown that the abundance of both Parkin and PINK1 mRNAs is reduced in response to mtDNA mutations (A3243G and T8993G) and total mtDNA depletion (rho^0^) which is also accompanied by a decrease in mRNA level for LC3 [72]. In primary fibroblasts from patients with MELAS (A3243G), Atg12, Beclin1 and LC3 are up-regulated at both the transcript and the protein levels and accompanied by a strong conversion of LC3-I into LC3-II [70]. Furthermore, microarray-based analysis of fibroblasts, lymphoblasts and myoblasts from Kearns–Sayre syndrome (KSS) patients, identified *Atg12* among the few up-regulated transcripts in response to mtDNA depletion [97]. Similar results were obtained for cybrid cells containing the large mtDNA deletion associated with KSS and the abundance of *Atg12* mRNA was even higher in mtDNA totally depleted cells (rho^0^) when compared with wild type counterparts [97]. It is worth mentioning that the disruption of the Atg12 (an ubiquitin-like modifier enzyme required for macroautophagy)-Atg3 complex increased mitochondrial abundance, fragmentation and reduced targeting of damaged organelles to autophagosomes upon CCCP treatment while lack of this complex has no impact on starvation-induced autophagy [98]. Finally, Atg12 has also been associated with mitochondrial apoptosis regulation by anti-apoptotic Bcl-2 and Mcl-1 protein inhibition [99]. Thus, it might be possible that Atg12 up-regulation shown in response to mtDNA alterations is associated with mitochondria-mediated apoptosis regulation. The consequences of this retrograde signalling on autophagy components at the protein level are not known yet and need further investigations. Indeed, the understanding of this retrograde signalling might be interesting in the context of mitochondrial myopathies to understand why altered versions of mtDNA accumulate and reach a high level of heteroplasmy, the ratio between altered and wild-type mtDNA abundance within a cell.

In conclusion, mitophagy has been shown to be mainly cyto-protective by removing partially depolarized mitochondria to prevent apoptosis induction and excessive ROS production, as demonstrated for ethanol-induced hepatocyte injury. Indeed, reduced hepatotoxicity and steatosis is associated with the specific degradation of both mitochondria and lipid droplets by autophagy in mice and cultured cells [100]. However, the possible link between autophagy, mitophagy, and lipid metabolism has only been demonstrated recently [20]. The next section will thus focus on current knowledge regarding the specific degradation of lipid droplets (lipophagy) and the crosstalk between autophagy and lipid metabolism.

## 3. Autophagy, Lipid Metabolism and Lipophagy

### 3.1. Autophagy and Lipids

Lipids are usually stored in cells as triacylglycerol molecules in small cytosolic structures called lipid droplets (LDs). Due to their complexity, these structures are now perceived as non membranous organelles that are divided in a core essentially made up of neutral lipids such as triglycerides (TGs) and of a unique monolayer containing several types of lipids (including cholesterol and glycerophospholipids such as phosphatidylcholine and lysophosphatidylcholine [101] in which several integral proteins are embedded [102]. The majority of these proteins belong to the “PAT” (Perilipin / Adipocyte Differentiation-Related Protein (ADRP) / Tail-Interacting Protein of 47 kDa (TIP47)) family. Two other proteins are also present in LD surface and share similarities with PAT proteins: S3–12 and OXPAT [102]. In this review, we will first briefly discuss the role of some of these proteins involved in the “classical” lipolysis. The reader who is interested in these proteins is invited to read the excellent review dedicated to the PAT protein family [102].

Perilipin A is the most abundant protein surrounding lipid droplets and represents a classical marker of adipocyte differentiation and regulator of TG content in the cells [103]. At the basal state, perilipin A is expressed in a non phosphorylated form. However, upon energy demand and appropriate cell stimulation, β-adrenergic receptors are activated, leading to the activation of PKA and the phosphorylation of perilipin A and the co-lipase CGI-58. This event allows the binding of HSL (Hormone Sensitive Lipase) to perilipin A and the recruitment of CGI-58 to ATGL (Adipose TriGlyceride Lipase) to stimulate lipolysis. These enzymes are involved in breakdown of triglyceride/triacylglycerol into diacylglycerol and in catabolism of this molecule into monacylglycerol, respectively. The final acyl chain is hydrolysed by the cytosolic monoacylglycerol lipase. Ultimately, the free fatty acids will be either re-esterified into triacylglycerol or will be used for mitochondrial energy production by the fatty acid β-oxidation pathway [104]. Under all conditions, the fatty acids are submitted to a rapid turnover by successive cycles of release/re-esterification inside the LDs. Even if this description of the “classical” or “canonical” lipolysis pathway has been accepted for years as the only way to degrade lipids and LDs, a recent study has pointed out the role of a lipid droplet-specific form of macroautophagy in lipid catabolism [20].

Although no clear link between lipids and macroautophagy was established until recent years, it was suspected for decades. Indeed, it has been shown that triacylglycerol lipase activity of isolated rat adipocytes seems to be confined to the acidic region of the pH curve (maximal activity at pH 4.4), data that suggested a role for lysosomal triacylglycerol lipase rather than cytosolic ATGL or HSL in this process [105]. Moreover, incubation of isolated adipocytes in the presence of lysosomotropic agents such as chloroquine (an inhibitor increasing lysosomal pH by still unclear mechanisms [106] might thereby inhibit lysosomal degradation and ultimately, at least under some specific conditions, fusion between autophagosomes and lysosomes [107]), ammonia or methylamine inhibits β-oxidation of fatty acids by 35 % [105]. Interestingly, this inhibition affected only endogenous fatty acid oxidation while catabolism of exogenous radioactive oleate was not affected by the treatment [105] This seminal study was further confirmed by another report showing that treatment using the same kind of inhibitors can inhibit the rate of ketone bodies formation in isolated 24 h-old rabbit hepatocytes, which could suggest a role for lysosomes in the breakdown of fatty acids [108].

**Figure 2:**
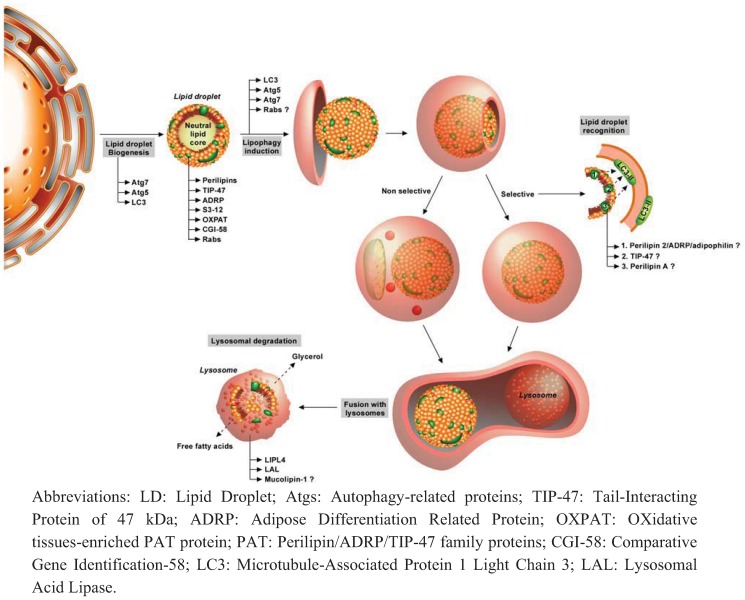
Mechanisms involved in lipophagy.

Lipid droplets are small organelles made up of a core of neutral lipid surrounded by a phospholipid monolayer in which several integral proteins (perilipins, TIP-47, ADRP, S3–12, OXPAT, CGI-58 and several members of Rab protein family) are embedded. Biogenesis of lipid droplets occurs in endoplasmic reticulum by budding of its membrane, even if the precise mechanism is still under discussion. Upon activation of autophagy such as starvation condition, lipid droplets can be degraded by autophagy either in a selective-process (lipophagy) or not (classical macroautophagy). Engulfment of lipid droplets by isolation membrane requires the presence of LC3, Atg5 and Atg7. A role for some Rab proteins has also been proposed recently. Selective degradation of lipid droplet would reasonably necessitate the presence of recognition proteins that would bridge the lipid droplet with LC3 proteins present in autophagosomal membranes. However, no clear demonstration of the existence of such proteins has been shown so far even if a role for perilipin A, ADRP/Adipophilin/perilipin 2 and TIP-47 has been suggested. Once the lipid droplet is completely engulfed in autophagosomes, this vesicle will maturate by fusion with lysosomes in order to form autophagolysosomes. This will ultimately lead to lysosomal degradation of the lipid droplet in order to release free fatty acids and glycerol, which can be either recycled or used for cellular energy production. Two lysosomal lipases have also been involved in lysosomal lipid degradation including LAL and LIPL-4. Presence of mucolipin-1 ion channel would also be needed for the acidification of lysosomal lumen and thus optimal lysosomal lipase activity.

However, the involvement of macroautophagy in lipolysis was not clearly demonstrated until Singh and his collaborators demonstrated the existence of a specific form of autophagy directed towards lipid droplets in the rat hepatocyte cell line, RALA255–10G (Figure 2) [20]. These authors showed for the first time that pharmacological inhibition of autophagy by 3-methyladenine (3-MA, a class III PI3K inhibitor [109]) clearly induces an increase in LDs population and in TG content in this cell line, with a maximal effect when incubated in the presence of exogenous lipids such as oleate. In addition, they also demonstrated that a knock-down of *Atg5* gene induces a similar effect. Culture in a methionine and choline-deficient medium induces a similar increase in TG content. Interestingly, Singh et al. also demonstrated a direct co-localization between typical markers of autophagosomes (LC3b) and LD staining (BODIPY) which suggests the existence of a specific form of autophagy towards lipids. These results were confirmed *in vivo*, as Atg7-deleted mice are characterized by an increase in TG content in the liver but not in other organs. In addition, the abundance of protein markers of LD such as TIP-47 and ADRP (adipocyte-differentiation related protein/adipophilin/perilipin 2) is also increased. Interestingly, they also show that LC3b-I is still localized on the surface of LDs in *Atg7* deficient mice, which therefore suggests that conjugation of phosphatidyletanolamine to LC3 is not required for association of this protein to LDs. In conclusion, this research group successfully demonstrated, in a major breakthrough, a specific form of autophagy towards LDs, called “macrolipophagy” to reference to the original non-specific macroautophagy [20]. However, one must keep in mind that this process may not be specific and might not be found in all tissues (Figure 2). Indeed, basal lipophagy does not seem to be a specific form as portions of LDs are engulfed together with other cytosolic components [110]. If cellular demand for lipid is sustained, the lipophagy process becomes finally selective, as the majority of autophagosomes contains only portions or entire LDs (Figure 2) [20,110].

Since this major publication, more recent studies have provided some additional details regarding the molecular machinery involved in the regulation of this specific form of autophagy. As already pointed out by Singh and colleagues, Atg7 seems to be crucial for lipophagy, as *Atg7*-deleted mice present evidence of inhibition of this process [20] a finding confirmed by others [111]. As already observed in mice, Atg7 loss of function mutant *Drosophila* also exhibits a decrease in number as well as in size of LDs [112], confirming its role in other species. Atg5 is also suspected of playing a role in lipophagy regulation, as *Atg5* knock out mice display defective adipogenesis and increased TG content [113]. Currently, beside the role of these two proteins, the implication of other Atg proteins remains relatively elusive.

However, as all the specific forms of autophagy discovered so far require specific molecular events, it is reasonable to think that lipophagy would also necessitate the presence of recognition protein(s) which would bridge the LDs and autophagy machinery. The lipid-associated protein TIP-47 could be one of the best candidates to play this role. Indeed, *Atg7* knock out mice present a considerably decreased TIP-47 abundance while the abundance of this protein is known to be independent of lipid content [20]. Therefore, TIP-47 could be degraded by autophagy and could make a link between LDs and macroautophagy machinery. Another interesting candidate could be perilipin 2/adipophilin as this protein co-localizes with LAMP-1 in mice macrophages [114]. Finally, the presence of LC3 seems also to be needed for lipophagy as its co-localization with both LD and lysosomal compartments has been described in numerous studies [20,114,115,116,117]. However, the role of these proteins in lipophagy is still poorly understood.

The role for the Rab proteins family in lipophagy has also been investigated. This protein family is composed of small GTPases that are involved in the regulation of intracellular trafficking including endocytosis, cytokinesis, autophagosomes formation, biogenesis of lysosomes and cellular signalling transduction [118]. Involvement of this protein family in lipid metabolism is not really a surprise as previous studies have already demonstrated the association of some members of this family such as Rab18 with LDs [119]. Recently, a screen to identify the putative role of of Rab GTPases in the regulation of LDs size has been performed in *Drosophila*. The authors found 14 Rab proteins involved in this process including Rab1, Rab5, Rab14, Rab21, Rab23, Rab27, Rab32, Rab40, RabX4, RabX6 (which increase the size of these organelles), Rab7, Rab10, Rab39 and RabX3 (having the opposite function) [112]. However, the interest of this study is limited to the comprehension of molecular mechanisms of recognition of LDs by lipophagy in other species than *Drosophila*, as some Rab proteins including RabX3, RabX4 and RabX6 have no counterparts in mammals. Interestingly, animals expressing negative dominant for Rab32 or perilipin 2 present the same phenotype, characterized by a reduced size of LDs [112]. The authors also observed a co-localization of Rab32 with autophagosome and/or lysosome markers, which suggests a potential role of this protein in autophagy [112]. In addition, in the liver of Rab32 mutant animals, the number of LC3-GFP puncta (clustered in autophagosomes) is decreased compared to wild-type liver mates, suggesting a defective autophagy in these individuals.

In conclusion, Rab32 seems to be involved in the regulation of LDs size by its function in autophagy, even if its precise role in this process is still unclear. However, we can exclude a direct link between this protein and LDs, as no co-localization between these two organelles has been described so far. Therefore, it would be of interest to study its role in mammalian cells. Another interesting candidate could be Rab18. Indeed, this protein that is essentially localized on the surface of LDs is recruited towards activation of lipolysis, following isoproterenol stimulation (a β-adrenergic receptor agonist), concomitant with fragmentation of LDs [119].

Although evidence for implication of autophagy in trapping and degradation of LDs has arrived in recent years, it remains unclear how the lipids are catabolised in lysosomes. Existence of lysosomal lipases has been well known for a long time but the precise role of these enzymes and thus their possible role in lipophagy is still questioned [110]. Indeed, it is classically admitted that lysosomal lipases are essentially involved in the catabolism of lipoproteins internalized by autophagocytosis [110]. However, a recent study has presented more details about the role of these enzymes in lipophagy. Incubation of mice peritoneal macrophages with acetylated low-density proteins (acLDLs) induces the formation of foam cells, which are very similar to those observed in atherosclerotic lesions [114]. These authors found that some LDs in these cells seem to be enclosed in acidic LAMP-1 positive vesicles (lysosomes) [114]. Adipophilin can also be found co-localized with these organelles. In accordance with original publication of Singh and colleagues, the authors also found a direct co-localization between LC3b and LDs. Electron micrographs also revealed that both the entire and parts of LDs are engulfed in double membrane structures similar to autophagosomes. Total protein LC3b-II abundance has also been found elevated in acLDLs exposed macrophages [114]. Taken together, these observations suggest the activation of lipophagy in these conditions. Moreover, the authors demonstrated that lysosomes could handle degradation of LDs in these cells as both E600, a non specific inhibitor of lipases that binds specifically to their active site, and chloroquine efficiently inhibit lipolysis and increase the cholesteryl esters content of macrophages exposed to acLDLs [114]. Concomitant with inhibition of autophagy process, LC3b protein abundance is increased by chloroquine. Interestingly, inhibition of lysosomal acid lipase (LAL) by lalistat can fully inhibit degradation of neutral lipids in acLDLs-treated macrophages and therefore increase the cholesterylester content in these cells. Finally, Atg5-deficient mice macrophages present a similar increase in cholesterylesters following treatment with acLDL [114]. Therefore, it seems that both neutral and lysosomal lipases (especially LAL) seem to be involved in lipophagy.

Another lipase candidate potentially involved in lipophagy could be Atg15p, which is the only Atg protein characterized by a lipase activity, at least in yeast as no homolog has been reported so far in mammals. This protein is localized at the endoplasmic reticulum membrane but can be rapidly recruited to the surface of autophagic bodies where it degrades the membrane of these structures [120]. Sequence analysis revealed that this enzyme could be involved in the degradation of neutral lipids such as TG [121]. Therefore, future work should be undertaken to address the role of Atg15p in lipophagy and to determine whether or not this protein is just involved in lipid maintenance of autophagosomes. Finally, the ion channel mucolipin 1 seems also to be required to maintain low lysosomal pH and lipase activity of this organelle [122].

### 3.2. Lipids and Autophagy

If autophagy can directly degrade some lipid cellular components, as LDs, lipids can also directly stimulate this process, especially during lipotoxicity. Lipotoxicity refers to the cytotoxic effects of excess fat accumulation in cells (other than adipocytes) and has been implicated as one of the contributing factors to diseases like obesity, diabetes and non-alcoholic fatty liver [123]. Indeed, these authors have shown that palmitate is able to rapidly induce (within 4 h) an increase in autophagy, as it elevates the LC3-II abundance and induces formation of LC3-positive foci in MEFs [123]. This effect seems to be independent of the inhibition of mTOR pathway as palmitate failed to reduce the phosphorylation of p70S6K, a direct target of mTOR kinase complex. Interestingly, only saturated fatty acids have been demonstrated as potent inducers of autophagy as oleic acid, an unsaturated fatty acid, has no effect on this process [123]. Nowadays, the advantages of lipid-induced activation of autophagy are still unclear. However, it has been reported that it could be involved in a pro-survival process in order to cope with the potent toxic effect of sustained presence of saturated lipid, a mechanism that would prevent/limit the deleterious effect of lipotoxicity. Indeed, the prolonged incubation of hepatoma cells (HepG2) or MEFs in the presence of palmitic acid induces the activation of PKCα that decreases cell death. The pro-survival role of this PKC isoform is also supported by the fact that the knock down of PKCα sensitizes cells to lipid-induced apoptotic cell death [123]. However, one must pay attention to the fact that other studies have reported controversial results about the role of lipids in the activation of autophagy. Indeed, incubation of INS1 cells (a pancreatic β-cell line) with 0.4 mM palmitate for 6 h suppresses autophagic turnover as evidenced by a strong inhibition of p62 degradation, an increase in LC3-GFP positive foci and the accumulation of autophagosomes [124]. Pancreatic β-cells of type 2 diabetes patients are also characterized by an accumulation of autophagosomes, which rather suggests a blockage of autophagy in these cells [125]. The negative impact of fatty acids on autophagy is also suggested by the fact that the fusion between autophagosomes and lysosomes is inhibited in the liver of high-fat diet mice. This inhibition has been attributed to modifications in the composition of autophagic compartment membranes [126].

The important interplay between autophagy, lipid metabolism and lifespan has been recently demonstrated in germline-less *C. elegans* [127]. Indeed, these animals present an increased expression of a predictive triglyceride lipase [128], LIPL-4/K04A8.5, caused by mTOR activation that induces the expression of two transcription factors: DAF-16 and PHA-4 [127]. Moreover, LIPL-4 overexpressing and germline-less animals showed an increase in autophagic flux, as reflected by an accumulation of glp-1 (an ortholog of mammalian LC3) positive foci and a reduced number of both autophagosomes and autolysosomes, accompanied by an increase in the protein abundance for Ulk-1, Beclin-1 and glp-1 [127]. Interestingly, knock down of these proteins inhibits the prolonged lifespan of LIPL-4 overexpressing animals, a finding that demonstrates a direct and close link between autophagy, lipid metabolism and lifespan, at least in *C. elegans* [127].

Although the link between lipid metabolism and autophagy is now accepted, interestingly no functional evidence of lipophagy does exist regarding its existence in adipocytes. First, it is possible that this form of autophagy could not occur in such cells, as they can rely on their high expression of different lipases (including ATGL and HSL) and expression of PATs proteins, which can be sufficient for regulation of lipid homeostasis. Another possibility is that lipophagy only occurs in adipocytes (and in other cells) in pathophysiological conditions such as obesity or other lipid-rich conditions. However this tempting hypothesis is challenged by the fact that lipid-induced triolein hydrolysis described in rabbit aortic foam smooth muscle cells cannot be affected by chloroquine, although this molecule could affect equivalently both cholesteryl esters and TG in untreated cells. This effect has been attributed to the difficulty of keeping a sufficiently low pH in lysosomes during lipid-rich conditions [129]. It is thus likely that prolonged incubation in the presence of lipids results in a detrimental effect on lysosomal pH that affects the lysosomal triglyceridase activity and, ultimately, inhibits lipid catabolism mediated by this organelle.

Therefore, it is possible that lipids could induce two different cellular responses depending on their composition, cell types, their effect on both lysosomal/autophagosomal membranes and their impact on lysosomal pH.

In adipocytes, instead of playing a role in lipid degradation, autophagy could rather regulate lipid metabolism in these cells mainly by controlling the adipogenesis process. Evidence for this hypothesis came from studies on *Atg5*-deleted MEFs. These cells are characterized by a dramatic reduction of adipogenesis as almost no more LDs (or only very few small droplets) can be seen in these cells, even after a long differentiation period [113]. Moreover, a few days after the induction of the differentiation program, cells that underwent adipogenesis, became TUNEL-positive (an assay detecting breakage of DNA molecules) and ultimately died [113]. Interestingly, mice invalidated for *Atg5* also present a reduced efficiency in adipogenesis and a decreased fat mass, observations that support the need for autophagy during adipogenesis, not only *in vitro* but also *in vivo* [113]. These results are consistent with other results obtained for *Atg7*-knock out mice. Indeed, a defect of adipogenesis is also observed in *Atg7*^-/-^ mice that might be responsible for a considerable drop in fat mass [111]. Furthermore, adipocytes of these mice are smaller, contain less LDs and are more often multivesicular than like adipose cells from wild-type littermates. Interestingly, these adipocytes present some characteristics of brown adipocytes such as a more abundant mitochondrial population and an increased fatty acid β-oxidation capacity [111]. However, basal and hormone-stimulated lipolysis is decreased in these cells. These two phenomena contribute to limit circulating fatty acids and lower plasma concentration, which could explain that adipocytes from *Atg7*^-/-^ mice are also more insulin-sensitive than wild-type adipocytes and why these animals are resistant to high-fat diet-induced obesity [111].

If autophagy directly regulates adipogenesis, the molecular mechanisms underlying this role are still unclear. The first hypothesis proposed that autophagy could regulate this process mainly by the control of LD biogenesis. Indeed, knock down of LC3b by RNA interference inhibits formation of LD in PC12 cell line but also in 3T3-L1 adipocytes [130]. Interestingly, LC3 can be recruited not only on the surface of the autophagosomes but also on the LD of starved hepatocytes and cardiomyocytes, where it directly co-localizes with both perilipin A and ADRP (Adipose Differentiation-Related Protein) [130]. Another study also reported the localization of LC3 on LD independently of the nutrient state, forming either a cup-shape or a ring-structure around the LD [115]. A co-localization between LC3 and perilipin A has also been described in adipocytes of obese patients [116]. Finally, the synthesis of LDs also requires Atg5/Atg7 as demonstrated in hepatocytes [117] but also in Atg5/Atg7 knock out mice [111,113].

### 3.3. Autophagy and Lipid Metabolism Disorders

Autophagy seems also to be a key process in some lipid metabolism disorders such as ethanol fatty liver and obesity. The first pathology is characterized by an excessive accumulation of lipid into the liver, due to heavy alcohol consumption. Alcoholic fatty liver is a potentially pathologic condition that can progress to steatohepatitis, fibrosis, and cirrhosis if alcohol consumption is continued and can also ultimately lead to cancer development. Although the molecular mechanisms involved in the development of this pathology are still unclear, it seems that lipid accumulation is not entirely caused by an increase in the *de novo* lipid synthesis [131]. A very recent study reports implication of autophagy in the development of this pathology. Indeed, acute ethanol-induced fatty liver of mice is characterized by the activation of PI3K, Akt and SREBP-1 (Sterol Regulatory Element-Binding Protein-1) [132]. Interestingly, pre-treatment of mice with wortmannin, a PI3K inhibitor, significantly decreases the lipid content of ethanol-exposed mice. Moreover, it correlates with autophagy activation (increased LC3II/I ratio and decreased p62 protein abundance) [132]. Therefore, these results suggest that (1) ethanol exposure can decrease lipophagy, even if it could be due to an indirect effect of increased lipid content, (2) lipophagy activation could decrease lipid content observed in these conditions and (3) lipophagy could rely on the activation of PI3K/Akt. The ethanol-induced macroautophagy has also been reported by another team for both mice liver and isolated primary murine hepatocytes [100]. However, only damaged mitochondria and lipid droplets seemed to be targeted by macroautophagy in these conditions, as degradation of long-lived proteins was not affected. Interestingly, antioxidants molecules such as NAC (N-acetylcysteine) or MnTBAP (Mn (III) tetrakis (4-benzoic acid) porphyrin chloride) and an inhibitor of alcohol dehydrogenase (4-MP : 4-methyl pyrazole), can effectively inhibit the autophagy activation in ethanol-exposed hepatocytes. Therefore, both ethanol metabolism and ROS production (that inhibit mTOR activity) are needed in order to activate autophagy in these conditions. Inhibition of autophagy in ethanol-treated hepatocytes also increases apoptosis, an observation that suggests that autophagy induced by ethanol is cytoprotective [100].

Another lipid metabolic disorder in which a role for autophagy has been described is obesity. This state is due to an imbalance between uptake and consumption of energy, which leads to hyperplasia and hypertrophy of adipose tissues, but also to ectopic fat deposits. The excessive accumulation of lipid in adipocytes induces mitochondria or endoplasmic reticulum dysfunction and at the end, to a global alteration of adipocyte biology leading to systemic modifications responsible for the metabolic syndrome and obesity-associated pathologies [133]. Until very recently, the potential implication of autophagy in the development of obesity was still largely unknown. Studies using the obese *ob*/*ob* mouse model have shown a decrease in the protein abundance of several key proteins of autophagy such as LC3, Beclin-1, Atg5 and Atg7, a process that could be due to activation of calpaïn 2 and suggests an inhibition of macroautophagy in obese mice. Concomitant with the inhibited autophagic flux hypothesis in these mice, the abundance of p62 protein is also higher in this model. Interestingly, in the obese mice, the inhibition of autophagy was correlated with the induction of ER stress and insulin resistance in murine isolated hepatocytes. This could be due to impaired insulin signalling as inhibition of *Atg7* expression results in reduced phosphorylation of Akt (Ser473) and phosphorylation of insulin receptor beta subunit [134].

However, it has been recently shown that the abundance of Atg5 and LC3 (a and b) are higher in omental adipose tissue of humans, when compared with their abundance in subcutaneous adipose tissue and, for a particular/given adipose tissue, increased in the adipose tissues of obese individuals when compared with tissues of non-obese individuals [135]. Interestingly, in a cohort of patients, the higher abundance of autophagic markers is correlated with insulin resistance [135]. These findings in patients suggest a link between autophagy, lipid metabolism and insulin sensitivity. Indeed, it has been reported that the activation of autophagy in adipocytes of obese patients could be due to attenuated mTOR signalling in response to insulin stimulation [116]. The lower activity of mTOR could lead to the co-localization of LC3 with perilipin A and could explain the increased lipolysis (by lipophagy) and mitophagy described in adipocytes of obese individuals, even if molecular events are still not clear [116].

ER-stress is also found in adipocytes of obese individuals that could lower adiponectin plasma concentration observed in these individuals, a process that could be mediated by the activation of autophagy. Interestingly, ER-stress relief or inhibition of autophagy restores adiponectin secretion in *db/db* and high fat-induced obese mice [136]. *In vitro*, ER-stress induced by thapsigargin in 3T3-L1 adipocytes also decreases the expression of insulin receptor by a mechanism that is dependent on autophagy [137]. Adipocytes from caveolin-deleted mice are also characterized by an increased autophagic flux (increased LC3-II/LC3-I ratio, decreased p62 abundance and decreased mTOR phosphorylation) [115]. Interestingly, caveolin-deleted adipocytes also exhibit decreased lipid mobilization in response to β-adrenergic stimulation and are insulin-resistant [115]. Therefore, autophagy seems to be a response allowing cells to face nutrient starvation eventually induced by caveolin knock out [115].

Obesity is also characterized by the activation of a sustained low-grade inflammation in adipose tissue but also on the whole body scale [138]. This inflammatory status has been associated with an altered adipokine secretion profile of adipocytes from obese patients, such as an increased IL-6 or TNFα secretion [139]. Recently, autophagy has been presented as a potent regulator of secretion of different pro-inflammatory adipokines and cytokines. Indeed, autophagy blockage by 3-methyladenine or bafilomycin A1 considerably increases IL-6, IL-1β and MCP-1 secretion in hypertrophic 3T3-L1 adipocytes [140]. Therefore, autophagy also seems to be a potent regulator (directly or indirectly) of pro-inflammatory mediators secretion by adipocytes. 

In conclusion, autophagy degrades cytoplasmic contents to achieve cellular homeostasis and, among these various contents, lipid droplets in a process called lipophagy. However, while active in some cell types such as liver cells in response to nutrient deprivation [20], it might not be a general phenomenon. Indeed, these authors have recently reported that selective loss of autophagy in hypothalamic proopiomelanocortin (POMC) neurons decreases α-melanocyte-stimulating hormone (MSH) levels, promoting adiposity, impairing lipolysis and altering glucose homeostasis. They propose that an unconventional, autophagosome-mediated, form of secretion in POMC neurons, regulates α-MSH production and secretion and thus eventually energy balance [141]. Since autophagy might affect the secretion of proteins and peptides, we end up this review by presenting recent findings about the role of autophagy in unconventional protein secretion.

## 4. Autophagy and Unconventional Protein Secretion

Most proteins that are secreted (estimated to 30 % of human genes encoding proteins) contain an N-terminal signal sequence that targets them to the ER/Golgi pathway [142]. While it was assumed that proteins which did not contain this targeting sequence were not secreted, recent proteomic studies on animal cells revealed that approximately 16% of the secreted proteins lack a signal sequence [143]. These findings strongly suggested that alternative secretion pathways must exist, called generically “non-classical” or “unconventional” secretion pathways. Currently, at least 20 mammalian proteins have now been clearly identified as being secreted through one of the multiple unconventional secretion mechanisms [144]. Among these proteins, one can mention FGF2 (fibroblast growth factor 2), a pro-angiogenic factor [145,146], insulin-degrading enzymes [147], the β-galactoside-specific lectins galectin 1 and 3, blood coagulation factor XIIIa, the engrailed homeoprotein, AcbA, an ACBP (Acyl-CoA binding protein), and several members of the interleukin family (IL-1 β, IL-18, IL-33) [148,149,150,151,152,153,154,155].

Even if not completely novel, since unconventional protein secretion was already reported 2 decades ago in yeast *Saccharomyces cerevisiae* for MAT (MAting Type regulatory protein), a factor secreted in an ER/Golgi-independent manner but mediated by an ATP-binding cassette (ABC transporter) encoded by *STE6* gene [156,157], an increasing number of proteins has been reported to be secreted by alternative pathways in eukaryotic cells [144]. Recent results highlight a novel role for autophagy that does not involve lysosomal degradation of autophagosomal content but instead involves a redirection of autophagosomes towards the extracellular delivery of an unconventional pathway for protein secretion. The role of autophagy in this process, evaluation of strength of evidence, implication for the field of protein trafficking and the future to fully establish autophagy in unconventional protein secretion have been recently reviewed [158].

The term “unconventional secretion” is thus referring to mechanisms by which proteins can be secreted by pathways other than the conventional secretory/exocytic transport route that goes through the ER, Golgi and TGN. Targeting of proteins to ER for the conventional secretion pathway involves N-terminal or internal sequences that are kept sequestered in coat protein complex II (COPII) vesicles nucleated by small GTPase Sar1 [159,160]. Many of these proteins also acquire a core N-linked glycosylation at the ER, and undergo subsequent modifications to their N-linked carbohydrate groups by glycosyltransferases when moving through the Golgi/TGN. The conventional secretion pathway also requires the GTPase ADP ribosylation factor 1 (Arf1) and coat protein complex I (COPI) complex [161].

At least two classes of proteins utilize non conventional transport to the cell surface: the first class contains proteins that harbour a signal-peptide sequence but reach the cell surface in a COPII- and Golgi-independent manner; while the second is secreted independently of both the ER and Golgi.

However, many unconventionally secreted proteins have been reported over the past two decades for proteins that neither have a signal sequence nor transit via the ER-Golgi route [154]. These alternative mechanisms involve cell surface membrane transport for proteins that are secreted but lack N-terminal secretion signal and protein modifications (mainly glycosylation since they fail to traffic thorough the ER/Golgi pathway) that characterize true transit through the secretory pathway [162]. Even if still debated, probably because numerous molecular effectors playing a role remain to be discovered, unconventional protein secretion appears to be based on several mechanisms allowing a classification into at least four groups belonging to either non-vesicular or vesicular categories: a direct translocation of proteins across plasma membranes by transporters, exosome secretion by fusion with the plasma membrane, plasma membrane blebs/microvesicles formation followed by shedding on the surface and eventually a participation of autophagy that requests uptake of proteins into endosomes or lysosomes (or a novel compartment with different markers) and subsequent fusion with the plasma membrane [144,154,158].

**Figure 3:**
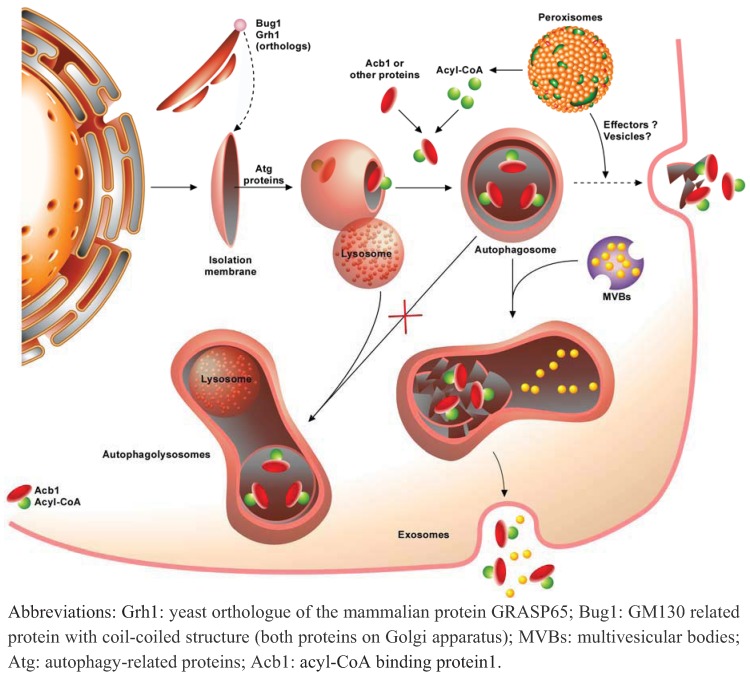
Mechanisms involving autophagy and unconventional protein secretion.

In yeast, a new vesicular mechanism has been described, suggesting the capture of a particular cargo such as Acb1 (an acyl-CoA binding protein1), a protein of 10 kDa processed on the cell surface by a cell membrane-associated trypsin-like prestalk protease (TagC) to generate a peptide with SDF-2 (Spore Differentiation Factor-2) like activity) involved in the induction of sporulation (Figure 3) [153,163]. The process would require the capture of the protein into autophagosomes that later on fuse with multivesicular bodies (MVBs)/or endosomes to form amphisomes which, in turn, fuse with the plasma membrane [164]. Indeed, the secretion of AcbA (an Abc1 ortholog) by *Dictyostelium discoidum* [153] and the unconventional secretion of Acb1 in *Pichia pastoris*, regulated and stimulated either by nitrogen starvation or rapamycin (that targets TOR and induces autophagy), is a process that involves GRASP (Golgi reassembly and stacking protein, not involved in starvation-induced autophagy) and autophagy machinery proteins such as Atg1, Atg6, Atg8, Atg9 and Atg17 [164]. These authors clearly show that Atg11, which is required for selective capture, receptor-dependent autophagy, was essential for Acb1 secretion (and thus SDF-2 like activity tested on the encapsulation of pre-spore of *Dictyostelium discoideum*) via autophagosomes. In Dictyostelium, AcbA was also found to accumulate below the plasma membrane just prior to secretion [165], a process not observed in mutants lacking key autophagy genes. In astrocytes, the maturation of ACBP by a trypsin-like activity at the cell surface leads to the production of endozepine peptides such as ODN and TTN, that modulate the sensitivity of the GABA receptor to γ-aminobutyric acid (GABA) [166].

In yeast, in which the process has been studied more, the exophagy would be responsible for the unconventional secretion of proteins as illustrated here for Acb1 protein. In response to starvation and nutrient privation, the autophagy, normally regulated by Atg proteins, might also have another fate than mediating subcellular compartment degradation. In these conditions, Acb1 protein would be packed and loaded into vesicles of autophagy that would be discriminated and use another pathway than fusion with vacuole/lysosome track. Although the mechanisms that deviate the autophagy vesicles containing the protein to be secreted are still poorly understood, the transport of Acb1-containing vesicle would change trafficking and end up by a fusion event with plasma membrane. This process could follow the still hypothetical route that would involve the fusion of autophagosomes and multivesicular (MVBs) leading to the formation of amphisomes and would be dependent on peroxisomes and the formation of Acyl-CoA that could play a role in the rerouting of the vesicle required for Acb1 secretion. Currently, a direct fusion between Acb-1 containing vesicle and plasma membrane cannot be excluded either.

By genetic analysis in yeast, Subramani’s group elucidated mechanisms by which the contents of autophagosomes ended up being secreted outside the cells. Indeed, using mutants for *VAM3*, encoding a vacuolar membrane protein involved in the fusion with autophagosomes; and for *YPT7*, encoding a small GTPase required for homotypic fusion during vacuole inheritance and endosome-vacuole fusion events and other mutants, they established that vacuolar delivery of autophagosomes and vacuolar turnover of autophagic bodies were not required for Acb1 secretion. However, proteins required for autophagosome closure (Tlg2), for vesicle fusion (Ypt6) and for the formation of MVBs (Vps4 and Vps23) are necessary for Abc1 secretion [163]. In addition, Acb1 unconventional secretion seems to be dependent on peroxisome-derived medium chain fatty acyl-CoA that are either binding Acb1 or necessary for Acb1 vesicle formation [164]. These authors clearly showed that yeast mutants lacking components of the classical ER/Golgi secretion pathway are able to secrete Acb1 protein normally while mutants affected in some autophagy genes failed to secrete the protein. Thus, the mechanisms involved in unconventional secretion of Acb1 require proteins necessary for the formation of autophagosomes that bypass the fusion with vacuole and any hydrolytic events and eventually fuse. This would lead to the formation of amphisomes (still to be characterized in yeast) that would eventually fuse with the plasma membrane, a process that involves the cell surface target membrane t-SNARE Sso1 and a phospholipase D (Spo14) [163]. While the detailed mechanisms by which the unconventional secretion of Acb1 might occur are still hypothesized, it seems that, as supported by genetic evidence, “exophagy” might play a crucial role in this process, even if some experimental data related to some crucial steps is still lacking in yeast [158,167].

However, since this process seems to be highly regulated and coordinated and not mediated by a direct fusion of autophagosomes with plasma membrane, a central molecular effector is most likely participating in the partitioning and the destiny of autophagosomes in the regular autophagic pathway and unconventional secretion of Acb1. Indeed, the group of Malhotra has recently identified Grh1, a protein in yeast *Saccharomyces cerevisiae* that localises into membrane structures near Sec13-containing ER exit sites. Through the recruitment of ESCRT (endosomal sorting complex required for transport) protein Vsp23 and the autophagy-related proteins Atg8 and Atg9, this structure, enriched in phosphatidylinositol 3-phosphate, might represent a “novel” compartment for unconventional protein secretion (CUPS) involved in the sorting of Acb1 [163,168].

Interestingly, while many of the proposed steps in the exophagy pathway in yeast still require biochemical and morphological characterization, the process of fusion between autophagosomes and endosomes and/or MVBs to generate amphisomes and fusion of amphisomes with the plasma membrane is better characterized in mammalian cells, although in these cells these pathways were not clearly associated with unconventional protein secretion (for a review [158]).

In addition, in mammalian cells, the role and mechanisms by which autophagy, already well characterized for autodigestive and quality control functions, contributes to biogenesis and secretion of IL-1β has been recently reported [169]. These authors have recently shown that the secretion of the pro-inflammatory cytokine depends on Atg5, inflammasome and some components of the GRASP paralogs, GRASP55 and Rab8a.

Thus, while mechanisms and molecular components of unconventional protein secretion are only beginning to emerge, they could include a role for caspase 1 and for the peripheral Golgi protein GRASP, which could function as a plasma membrane tether for membrane compartments during specific stages of development [144]. In addition, to date, no consensus model for the origin of the isolation membrane in mammals has been reached, although endosomes, mitochondria, ER and the Golgi have all been suggested as possible membrane providers for the formation of autophagosomes [170], it is still thus most likely that autophagosomes from different origins, bearing various markers would be used by cells to sort and secrete proteins by unconventional secretion pathways. 

One can still mention that as ACBPs (Acyl-CoA binding proteins) share a high degree of conservation between *H. sapiens*, *P. Pastoris*, *S. cervisiae*, it is likely that the secretion of these ACBPs follows the same route taking the exophagic pathway. While mechanisms linking autophagy machinery to protein secretion are only starting to emerge, the role of (dys)regulation in physio-pathological situations is still in its infancy. However, as ACBP is the precursor of endozepine peptides synthesised in astrocytes that could modulate the GABA_A_ receptor [166], the role of exophagy and alterations in this pathway, especially in neurological disorders, remains to be fully explored in humans.

## 5. Conclusions

Autophagy has been extensively studied during recent decades and thousands of papers have described the existence of more than 30 autophagy-related proteins. Moreover, three different types of autophagy have been discovered so far including macroautophagy, microphagy and chaperone-mediated autophagy. However, it is only during the very recent years that the implication of autophagy in many cellular processes has been reported including: Molecules recycling and cellular energy production, regulation of cellular differentiation, balance between cell death (apoptosis, necrosis, necroptosis, and pyroptosis) and survival (e.g., facing nutrient starvation), regulation of the immune system, and implication in the ageing and tumorigenesis process. Although autophagy was initially considered as a non-selective process, a lot of studies have produced details concerning specific forms of autophagy for several organelles or cellular compartments, including mitochondria, endoplasmic reticulum, peroxisosomes or poly-ubiquitinated proteins. In this review, we have first summarized the more recent findings about the mitophagy process and more specifically its implication in mitochondrial dysfunction conditions as well as in the general interplay and crosstalks between mitochondria and autophagy. If great advances in characterization of molecular mechanisms have been made in recent years, some questions remain unanswered such as the precise consequences of mutations of mtDNA on autophagy and mitophagy, as well as the role of Atg12 up-regulation in this context and its impact on the autophagy process. More data are also required in order to understand the precise role of endoplasmic reticulum stress on mitochondrial population and putative subsequent mitophagy control.

In the second part of this review, we have detailed the more recent findings about the lipid-droplet-specific form of autophagy, lipophagy. For this even more recently discovered biological response, several questions also remain almost completely unanswered. What is (are) the protein(s) (if any) involved in the recognition of lipid droplets by autophagy machinery? Is this machinery completely the same as that used for classical macroautophagy or does it rely on other dedicated proteins? We also have to mention that we still do not know whether this process does exist in every cell type or not. Indeed, clear evidence for the existence of lipophagy in adipocytes is still lacking. Therefore, it would be of interest to evaluate this in future studies. Furthermore, extensive study of the putative implication of lipophagy in lipid-associated diseases such as cardiovascular diseases would also be very important.

Finally, we discussed the role of autophagy in unconventional secretion of proteins and peptides. Although we have presented the first details known about molecular machinery involved in this pathway, it remains clear that further studies are needed in order to obtain a better understanding of this process, especially in mammalian cells as major studies were undertaken in yeast strains. Furthermore, it will also be necessary to determine if unconventional secretion of proteins relies on a common and shared molecular machinery or on a protein-specific machinery. One of the best candidates would certainly be the ACBPs (acyl coenzyme A (acyl-CoA) binding proteins) as a high degree of conservation of such proteins among species is observed.

In conclusion, autophagy is an important process as it is involved in numerous cellular processes, as described above. However, a lot of questions remain unanswered and responses would help in order to obtain a better understanding of cellular biology. This is needed for the use of autophagy as a potential target for new therapeutic strategies in disorders in which a deregulation of autophagy might play a role including pathologies associated with mitochondrial dysfunction and lipid-associated disorders.
